# 3D numerical modeling of the deformation and failure mechanisms of batter pile groups subjected to fault ruptures

**DOI:** 10.1038/s41598-024-83044-9

**Published:** 2025-01-23

**Authors:** Mukhtiar Ali Soomro, ZiQing Zhu, Muhammad Shamrooz Aslam, Hazrat Bilal, Abid Yahya, Irfan Anjum Badruddin

**Affiliations:** 1 School of Mechanics and Civil Engineering, China University of Mining and Technology, Xuzhou, 221116 Jiangsu People’s Republic of China; 2 Artiffcial Intelligence Research Institute, China University of Mining and Technology, Xuzhou, 2211106 People’s Republic of China; 3 College of Mechatronics and Control Engineering, Shenzhen University, Shenzhen, 518000 People’s Republic of China; 4 Department of Electrical and Communications Systems Engineering, Botswana International University of Science and Technology, Palapye, Botswana; 5 Department of Mechanical Engineering, College of Engineering, King Khalid University, Abha, 61421 Saudi Arabia

**Keywords:** Fault rupture, Battered pile foundation, Earthquake, Numerical modelling, Civil engineering, Software

## Abstract

Fault ruptures induced by earthquakes pose a significant threat to constructions, particularly underground structures such as pile foundations. Among various foundation types, batter pile foundations are widely used due to their ability to resist inclined forces. To gain new insights into the response of batter pile groups to fault ruptures caused by earthquakes, this study investigates the deformation and failure mechanisms of batter pile groups due to the propagation of normal and reverse fault ruptures using 3D numerical modeling. An advanced hypoplastic constitutive model for clay, which accounts for small-strain stiffness, and a concrete damage plasticity (CDP) model are employed to simulate the soil and the batter pile foundation, respectively. Results show that following fault propagation, nearly 10% tilting and significant displacement occurred at the pile cap, indicating a total failure of the batter pile foundation. It was also observed that the piles bent towards the slipping direction of the hanging wall. Tensile damage to the pile foundation was notably more severe than compression damage. The most severely damaged regions were not only located at the joints between the piles and the pile caps but were also found along the pile shafts.

## Introduction

Batter pile foundations are commonly employed to resist tilt and lateral movement in superstructures such as bridge piers and transmission towers^2,12,13,53^. Despite their ability to effectively resist horizontal forces and dynamic loads induced by earthquakes, post-seismic observations from major earthquakes have indicated that pile foundations may be less effective than stiff mat foundations in protecting structures against significant fault ruptures beneath them^7–10,29,31^. Additionally, many building codes and standards discourage the use of batter piles in seismic regions due to several issues, including the large forces induced onto the pile cap, the reduction in bending capacity due to axial forces, unfavorable cap rotations, and residual bending moments caused by soil settlement before an earthquake^5,6^. Fault ruptures induced by earthquakes often result in substantial ground movements in various directions, posing significant risks to foundations^4,14–18^. While extensive research has been conducted to investigate the behavior of pile foundations under fault ruptures, the interaction between batter pile foundations and soil remains inadequately understood^4–10,12−20,30^. Centrifuge model studies and numerical simulations have been performed to observe the interaction between fault ruptures and different types of pile foundations^7,14,15,17,34–36,44,56^. Related research, such as the interaction between soil movement and underground structures, has also been extensively studied. These studies have revealed tilting, deformation, and significant internal forces that can severely compromise the bearing capacity of structures. However, normal and reverse fault ruptures have distinct effects on soil and structures, and limited research has examined their respective impacts on pile foundations. To better understand the propagation of fault ruptures from bedrock to the ground surface, finite element analyses and sandbox tests have been conducted^6,21,23,27,36–38,44,46,47,50^. These studies have investigated the propagation and geometric characteristics of fault ruptures in detail. It has been found that the offset of the bedrock (i.e., the fault distance) is a critical factor influencing fault rupture propagation. Longer fault distances often result in more severe soil movements, which can significantly damage underground structures such as tunnels and pile foundations. Studies in the literature have demonstrated that shallow fault ruptures can significantly impact geotechnical structures due to the amplification of displacements and stresses near the surface. Several studies have modelled shallow fault ruptures to investigate their influence on geotechnical systems. For instance, Bray et al.^16^ and Paolucci et al.^45^ explored the effects of fault-induced displacements on shallow foundations and retaining structures, explicitly emphasizing the importance of understanding near-surface faulting effects. These studies provide a precedent for analyzing the interaction of shallow fault ruptures with engineered structures. Ng et al.^44^ demonstrated fault-rupture propagation in uncemented and cemented clay using centrifuge tests, highlighting the development of shear zones at shallow depths. Cai and Ng^17^ showed that shallow fault movements significantly affect pile foundations, inducing bending moments and settlement even for piles embedded at moderate depths.

The observation about the relatively shallow embedment of the pile group (less than 19 m) is accurate. However, shallow fault ruptures are often associated with complex ground deformations that can significantly impact piles at this depth. Numerous researchers have shown that shallow rupture propagation can create localized shear zones that significantly impact piles and shallow foundations^1,10,43,45^. The interaction between shallow foundations such as rafts and isolated footings and normal and reverse fault ruptures has been studied extensively using numerical simulation^6,10,36^, experimental modelling^1,14,15,43^, and field evidence from recent earthquakes^4,5,20,54^. Bransby et al.^14,15^ used centrifuge modelling to study the interaction between normal and reverse fault ruptures and strip foundations. They found that the foundation bearing pressure, the thickness of the soil, the relative location of the raft to the free-field outcrop, and the width and rigidity of rafts has a huge impact on the geotechnical performance of foundations subject to fault rupture. Ahmed et al.^1^ carried out centrifuge tests to assess the interaction between the reverse fault rupture and shallow foundations and concluded that their response to reverse faults is similar to normal faults, and is a function of the relative distance between a fault outcrop and the foundations. Faccioli et al.^20^ compared their field evidence from recent earthquakes with numerical simulation and showed that structures that rest on flexible foundations such as isolated foundations could experience structural damage ranging from partial to full collapse, depending on the severity of differential settlement at the ground surface. However, a structure sitting on a rigid foundation or raft and caisson foundations that is subjected to the substantial settlement at the surface could resist applied large amount of structural distress^4,5^. To enhance the performance of pile foundations under fault rupturing, a novel foundation system incorporating cushioned piles beneath the raft was proposed and evaluated using Abaqus. The results demonstrated that this innovative cushioned pile raft foundation effectively mitigates the impact of ground movements on the structure, making it a viable solution against fault ruptures. Additionally, another foundation system featuring a geosynthetics-reinforced interposed layer was also proposed. Simulation results in Abaqus indicated the superior performance of this composite foundation system, showing a significant reduction in shear forces within the raft and piles.

Most previous studies have focused on the effects of fault rupture on shallow or vertical pile foundations. Consequently, there is a lack of systematic research on the responses of batter pile groups subjected to vertical loads during the propagation of normal and reverse fault ruptures. This study addresses this gap by investigating the deformation and failure mechanisms of batter pile groups under these conditions using 3D numerical modeling.

## Characteristics of finite element analysis

### Description of geometry of the numerical models

**Fig. 1:**
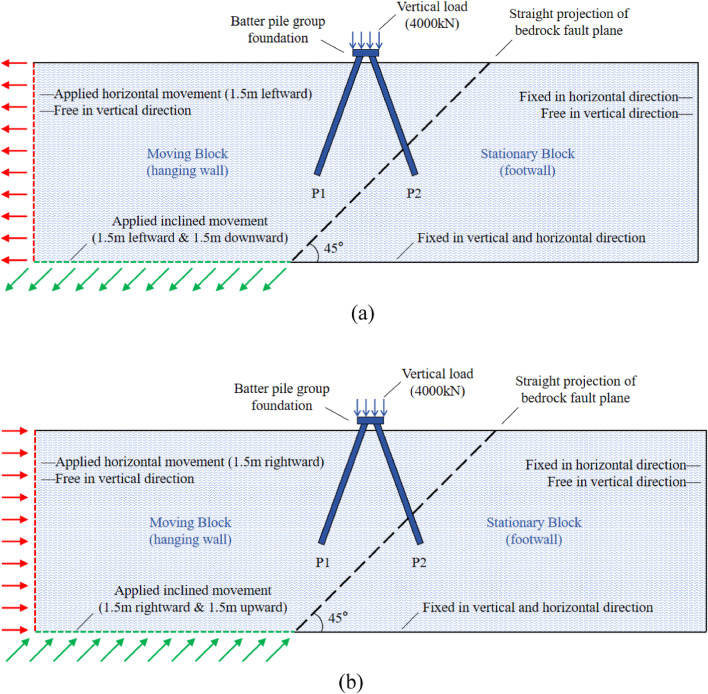
Schematic representation of the generation of the fault ruptures (**a**) normal fault (**b**) reverse fault.

This hypothetical study explores the interaction effects of fault ruptures (both normal and reverse) with a (2 × 2) batter pile group embedded in stiff clay, under scenarios caused by the movement of the underlying bedrock^18–24^. To achieve this, three-dimensional numerical analyses were performed using the finite element software Abaqus 6.14-2 (Hibbitt et al.^25^). Since fault rupture events occur very rapidly, undrained analyses were conducted to evaluate their effects on the battered pile group. Figure 1a and b illustrate the geometry of the numerical models for normal and reverse fault ruptures, respectively. These models examine the behavior of a (2 × 2) batter pile group subjected to vertical loading under different faulting conditions. Each pile in the group has a diameter of 0.8 m and an embedded length of 18 m. The four piles are connected by a 4 m × 4 m × 1 m pile cap, with a center-to-center spacing of 2 m between piles at the cap level. All piles are inclined at a batter angle (*θ*) of 20°, which is considered an optimized angle for batter piles based on previous studies^12,24,53^. In the numerical analysis, the pile installation effects on the in-situ stress distribution of the soil were not considered. Consequently, the piles were modeled as “wished-in-place,” which assumes their behavior closely resembles that of bored piles. While this approach simplifies the modeling, it may result in conservative estimates of the computed responses. The pile group was reinforced with steel bars. Each pile was reinforced with six 20 mm diameter longitudinal bars, equally spaced around the pile. These were connected with 10 mm diameter spirals at 200 mm center-to-center spacing. The pile cap reinforcement consisted of 25 mm diameter bars, spaced at 200 mm in both the *x* and *y* directions. A vertical load of 4500 kN was applied on pile cap. Based on predictions of fault propagation^50,56^, the batter pile foundation was strategically placed near the anticipated fault plane to ensure that the ruptures would intersect with the pile group, as illustrated in Fig. 1. This positioning allows the study to evaluate the performance of the foundation under realistic faulting conditions.

### Geometry discretization, boundary and initial conditions

**Fig. 2:**
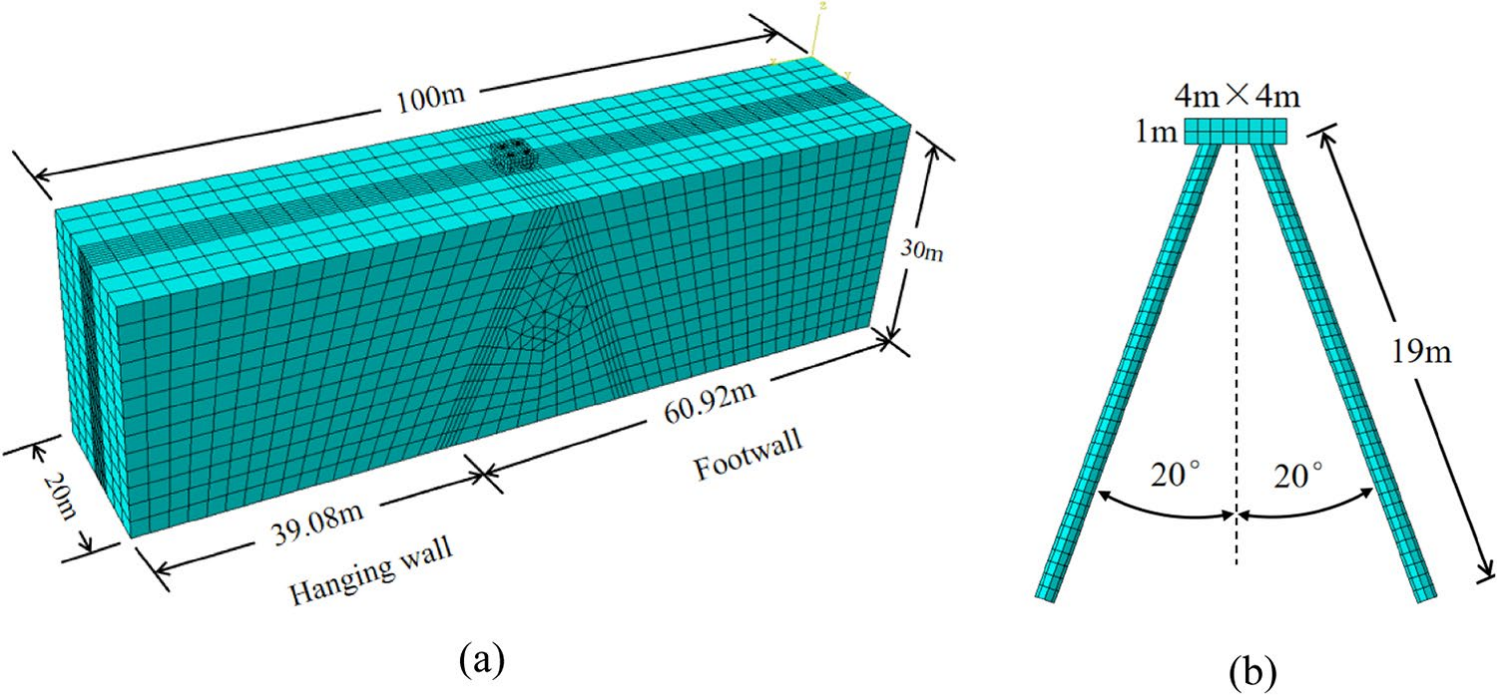
Finite element mesh of the numerical analysis: (**a**) overview (**b**) batter pile group foundation.

The primary objective of this study was to utilize three-dimensional finite element models to accurately represent the behavior of battered piles in response to fault ruptures. Figure 2a provides an isometric view of a typical finite element mesh for the normal fault scenario. The dimensions of the mesh are 100 m in length along the *y*-axis, 20 m in width along the *x*-axis, and 30 m in depth along the *z*-axis. The mesh consists of 27,676 elements and 27,094 nodes. The soil domain was discretized using 8-node brick elements with trilinear displacement and trilinear pore pressure capabilities. Similarly, the piles were modeled using 8-node linear brick elements, as shown in Fig. 2b. To represent the steel reinforcements in the piles and the pile cap, two-nodded truss elements were employed.

During the steps for generating geostatic stresses and installing the battered pile group, all displacement components were fixed to zero at the base of the mesh, while only displacements perpendicular to the vertical boundaries were constrained. Geostatic (in-situ) stresses were initialized using a saturated bulk unit weight of 20 kN/m³ and a hydrostatic pore pressure distribution along the depth of the model with a coefficient of lateral earth pressure, Ko. Once equilibrium was achieved, the elements representing the pile group were activated within the soil mesh. Subsequently, a vertical load) was applied at the top of the pile cap. After the stabilization of effective stresses due to consolidation under the applied load, fault rupture simulation was initiated. Since the primary focus of this study is the interaction mechanism between fault rupture and the foundation, the bedrock was modeled as a rigid boundary. To simulate fault rupturing in the bedrock, the bottom boundary corresponding to the bedrock surface was divided into two parts, as shown in Fig. 1. The left part represented the hanging wall (the moving block), while the right part simulated the footwall. During fault rupture simulation, the hanging wall boundaries moved downward parallel to the dip angle (i.e., α = 45°). To satisfy equilibrium, the bottom and left boundaries of the hanging block were subjected to displacement to simulate fault movement. For reverse fault scenarios, the boundary conditions were reversed. The hanging wall boundaries moved upward at a dip angle of 45° to the horizontal plane, causing reverse faulting^46,47^.

An essential aspect of modeling soil-structure interaction (SSI) is establishing realistic interactions between the pile and the surrounding soil. Relative movement between the pile and the soil, as well as potential separation between the raft and the soil, can occur during fault rupture propagation. To incorporate these interactions, the surface-to-surface contact technique provided in the Abaqus software package was used. The interface was modeled using Coulomb friction law, which requires the interface friction coefficient (*µ*) and a limiting displacement (*γ*_*lim*_) as input parameters. A limiting shear displacement of 5 mm was assumed to achieve full mobilization of the interface friction, calculated as *µ* × *p’* is the normal effective stress between the two contact surfaces. A typical value of *µ* = 0.35, appropriate for bored piles, was used in all analyses^50–52^.

### Constitutive models and parameters

The hypoplastic model can simultaneously reflect the nonlinear stress-strain behaviour of soil during loading and unloading, and it is an effective constitutive model for describing the mechanical behaviour of soils^22,41^. Compared to other models based on elastoplastic theory, the hypoplastic model can only calculate the total strain, therefore the most significant advantage of this constitutive model is that the model can be independent of yield surfaces and other elastoplastic-related components. In other models, the type and location of potential sliding surfaces can be represented by plotting the equivalent plastic deflection strain, however, in the hypoplastic model, the potential sliding surfaces can be represented by the distribution of critical friction angles. According to the formula summarized by Mašín^40^ the general form of the hypoplastic model is shown in Eq. (1).

1 $$\dot{\sigma } = f_{s} \left[ {L(\sigma ,e):\dot{\varepsilon } + f_{d} N(\sigma ,e)\left\| {\dot{\varepsilon }} \right\|} \right]$$

where $\dot{\sigma }$ denotes the stress ratio tensor; ${\dot{\varepsilon }}$ denotes the strain rate tensor; *L* and *N* are the fourth-order tensor function and the second-order tensor function representing the state of the soil body, respectively.$(L(\sigma ,e):\dot{\varepsilon })$is a linear term that indicates a linear increase in stress with respect to strain. The second part $(N(\sigma ,e)\left\| {\dot{\varepsilon }} \right\|)$ establishes a nonlinear relationship between the stress increment and the strain increment. The stiffness factor *f*_*s*_, which controls the effect of the mean effective stress, can be determined from isotropic compression, which reflects the influence of barotropy, and *f*_*d*_, which is a densification factor related to the pore ratio, which reflects the influence of pyknotropy. This was further modified to make the model capable of predicting the response of clay due to monotonic and cyclic loading at small, medium, and large strain levels, and improving the performance of the model in the small strain range^39,40,57^. Stiffness anisotropy is also added to further improve the model.

The hypoplastic model of clay used in this study contains a total of 14 model parameters. Four of the parameters, *φ*_*c*_*’*, *N*, λ*, κ* are the same as those defined in the generalized Cambridge model. The parameter *φ*_*c*_*’* is the effective friction angle of the clay in the critical state, and the parameters *N*, λ*, κ* are the onset of the isotropic compression line (NCL) in the *ν* - ln*p’* space, the slope of the isotropic compression curve, and the slope of the unloading line (the instantaneous bulk modulus), respectively, where *p’* is the mean effective stress. In addition to the four parameters controlling the monotonic behaviour of clay at moderate to large strain levels, there are six other parameters used to control the small-strain stiffness (the concept of intergranular strain) of clays under different strain path inversions (related to the response of the soil under cyclic loading), *R*, *β*_*r*_, *χ*, *A*_*g*_, *n*_*g*_, and *m*_*rat*_. *R* stands for the strain range of the elastic strain in the soil; *β*_*r*_ and *χ* control the strain dependence of the soil stiffness under small strain conditions (the rate of decrease of the soil stiffness with strain); *A*_*g*_ and *n*_*g*_ control the stress dependence of the soil stiffness under small strain conditions. The parameter *m*_*rat*_ is obtained by dividing *m*_*T*_ and *m*_*R*_, which are two scalar multipliers used to correct the sub plastic tensor *L*(*σ*,*e*) and *N*(*σ*,*e*), which control the path dependence of the soil stiffness under small strains. *ν*_*pp*_ controls the ratio of volume and shear stiffness, replaces the parameter *r* in former studies, and is calibrated using the same process as parameter *r*. The parameter *ν*_*pp*_ is defined as the ratio of the volume and shear moduli in the isotropic plane. where the subscript *p* denotes the orientation in the isotropic plane, usually indicating the horizontal direction, and is defined as the ratio of the volume to the shear modulus for a test starting from an isotropic normal compressive stress state. The parameter *p*_*t*_ denotes the average stress change due to cohesion and replaces the effective stress *σ* in the model equation with *σ* - *p*_*t*_. The parameter *α*_*G*_ represents the ratio of the horizontal shear modulus to the vertical shear modulus.

Prior to carrying out numerical analyses, it is quite important to calibrate and validate the model parameters against measured results obtained from laboratory tests. The parameters of the hypoplastic clay model for kaolin clay were used in this parametric study because the extensive amount of laboratory tests performed on kaolin clay to obtain its geotechnical properties are available in the literature^3,11,22^. Hong et al.^26^ calibrated hypoplastic clay model parameters for kaolin clay against experimental results. The parameters (hypoplastic clay model) for kaolin clay used in this study were adopted from the numerical study reported by Wang et al.^55^. The overly consolidated clays are subjected to pre-consolidation stress in the past higher than the current pressure due to unloading or aging effects. The coefficient of lateral earth pressure at rest (K_0_) highly depends upon the over consolidation ratio^49^.

**Fig. 3:**
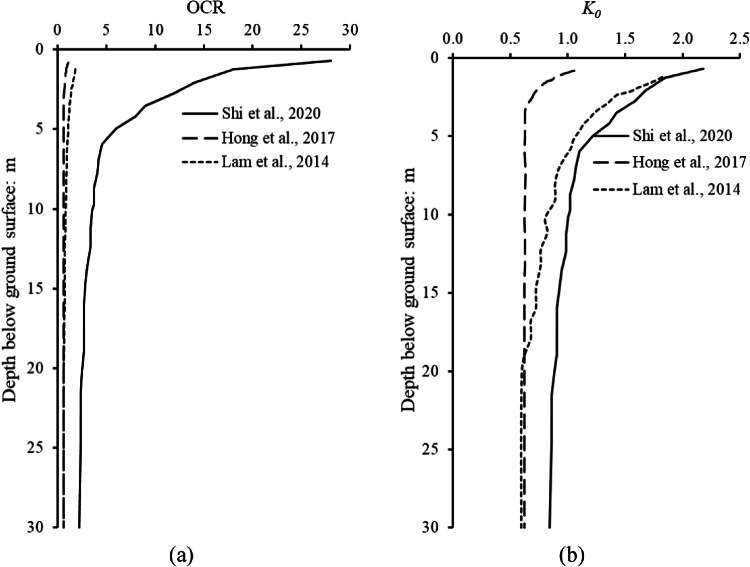
(**a**) Distribution of over-consolidation ratio; (**b**) Deduced K_0_ profile of clay used in all analyses.

Figure 3a illustrates the OCR profile along the depth measured in centrifuge tests performed by Shi et al.^48^, Hong et al.^26^, and Lam et al.^33^. The OCR profile decreased along with the depth below the ground surface. They obtained effective unit weight of clay by measuring soil water content after completing high-g consolidation. By considering the applied vertical effective stress at clay surface during high *g*-level consolidation, OCR values along soil depth were calculated. As expected, the maximum values of OCR occurred at the ground surface, and it decreased rapidly with soil depth. Figure 3b compares the deduced K_0_ profiles along with the depth below the ground surface from OCR values. The K_0_ is estimated by Mayne and Kulhawy^42^’s equation which correlates K_0_ and OCR. Since the constitutive model parameters were validated against centrifuge test (performed in stiff kaolin clay), the OCR and corresponding K_0_ profile by Shi et al.^48^ were adopted in this study. The values of all parameters of the hypoplastic clay model for kaolin clay used in this parametric study are given in Table 1.

**Table 1:** Parameters of the hypoplastic model used in the numerical simulations.

Parameter	Value	Reference
Critical state friction angle, *φ*^′^_*c*_	22^°^	Wang et al.^55^
Parameter controlling the slope of the isotropic normal compression line in the ln(1 + *e*) versus ln*p* plane, λ^*^	0.095	Deduced from Al-Tabbaa^3^
Parameter controlling the slope of the isotropic unloading line in the ln(1 + *e*) versus ln*p* plane, κ^*^	0.015	Deduced from Al-Tabbaa^3^
Parameter controlling the position of the isotropic normal compression line in the 1n(1 + *e*)-ln*p* plane, *N*	1.344	Deduced from Al-Tabbaa^3^
Parameter controlling the shear stiffness at medium- to large-strain levels, *r*	0.5	Wang et al.^55^
Parameter controlling the initial shear modulus upon 180^°^ strain path reversal and in the initial loading, *m*_*R*_	9	Calibrated based on Benz^11^
Parameter controlling the initial shear modulus upon 90°strain path reversal, *m*_*T*_	11	Calibrated based on Benz^11^
Size of the elastic range, *R*	1e^− 5^	Calibrated based on Benz^11^
Parameter controlling the rate of degradation of the stiffness with strain, *β*_*r*_	0.1	Calibrated based on Benz^11^
Parameter controlling the rate of degradation of the stiffness with strain, *χ*	1	Calibrated based on Benz^11^
Dry density (kN/m^3^)	1614.7	Deduced from measured void ratio
Permeability, *k* (m/s)	1e^− 9^	Al-Tabbaa^3^

**Fig. 4:**
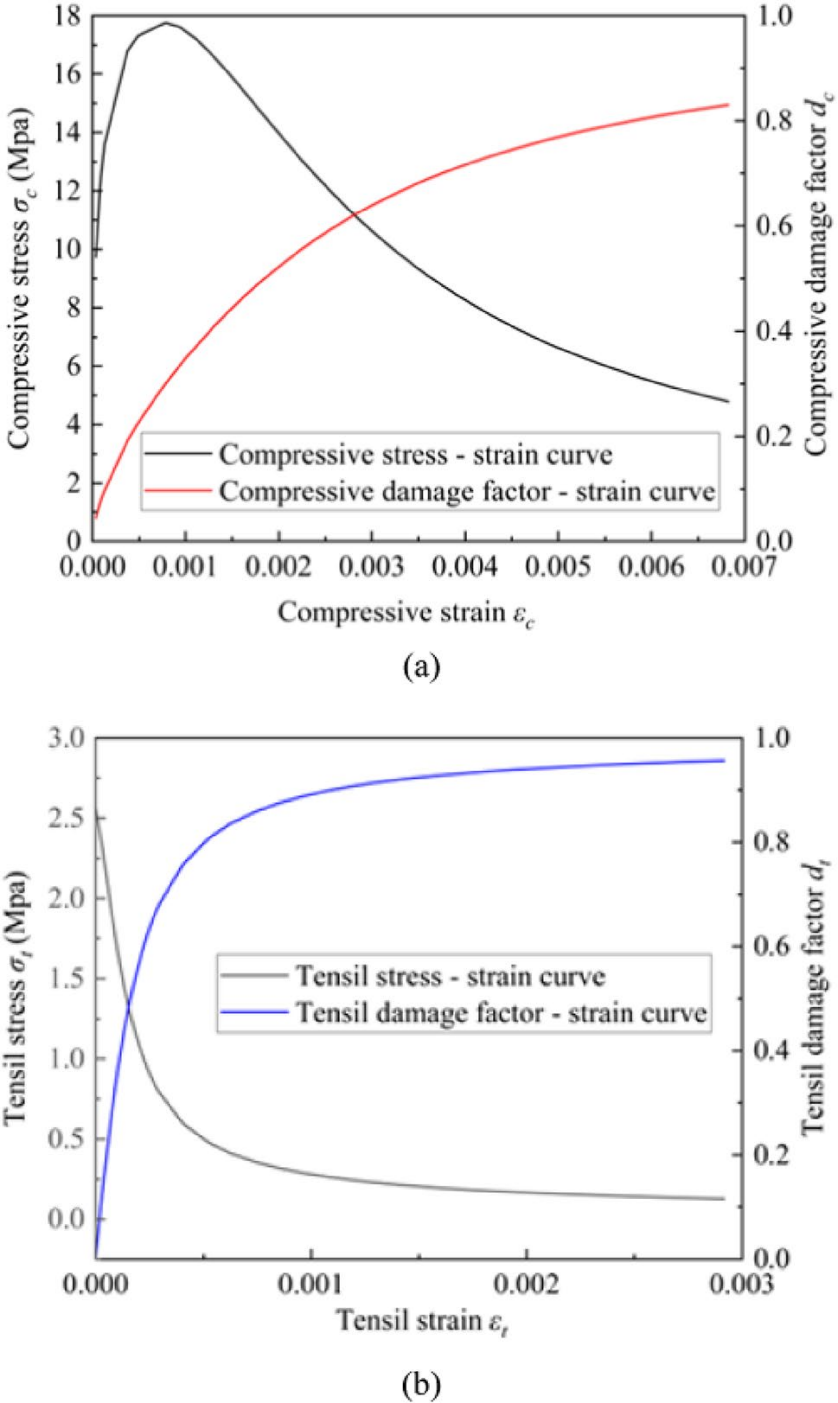
Stress-strain relationship and damage factors of C35 concrete in (**a**) compressive mode (**b**) tensile mode.

The pile in this study is modeled using a concrete damage plasticity model, with the concrete grade as C35. The basic parameters are provided in Table 2. Figure 4 illustrates the development curves of uniaxial compressive stress and damage factor with strain, as well as uniaxial tensile stress and damage factor with strain. The stress-strain behaviour is described using the damage factor and plastic strain, showing that both the elastic modulus and strength decrease as plastic strain increases.

**Table 2:** Parameters of concrete damage plastic model.

Description	Parameter	Value
Elastic modulus (GPa)	*E*_*p*_	30
Poisson’s ratio	*υ*	0.2
Mass density (kg/m^3^)	*ρ*	2500
Dilation angle (°)	*ψ*	38
Eccentricity	∈	0.1
Ratio of ultimate strength under biaxial compression to uniaxial compression	*f*_*b0*_ / *f*_*c0*_	1.16
Invariant stress ratio	*K*	0.667
Viscosity	*µ*	1e^− 5^
Standard value of compressive strength (MPa)	*F*_*ck*_	25.32
Standard value of tensile strength (MPa)	*F*_*tk*_	2.57

**Fig. 5:**
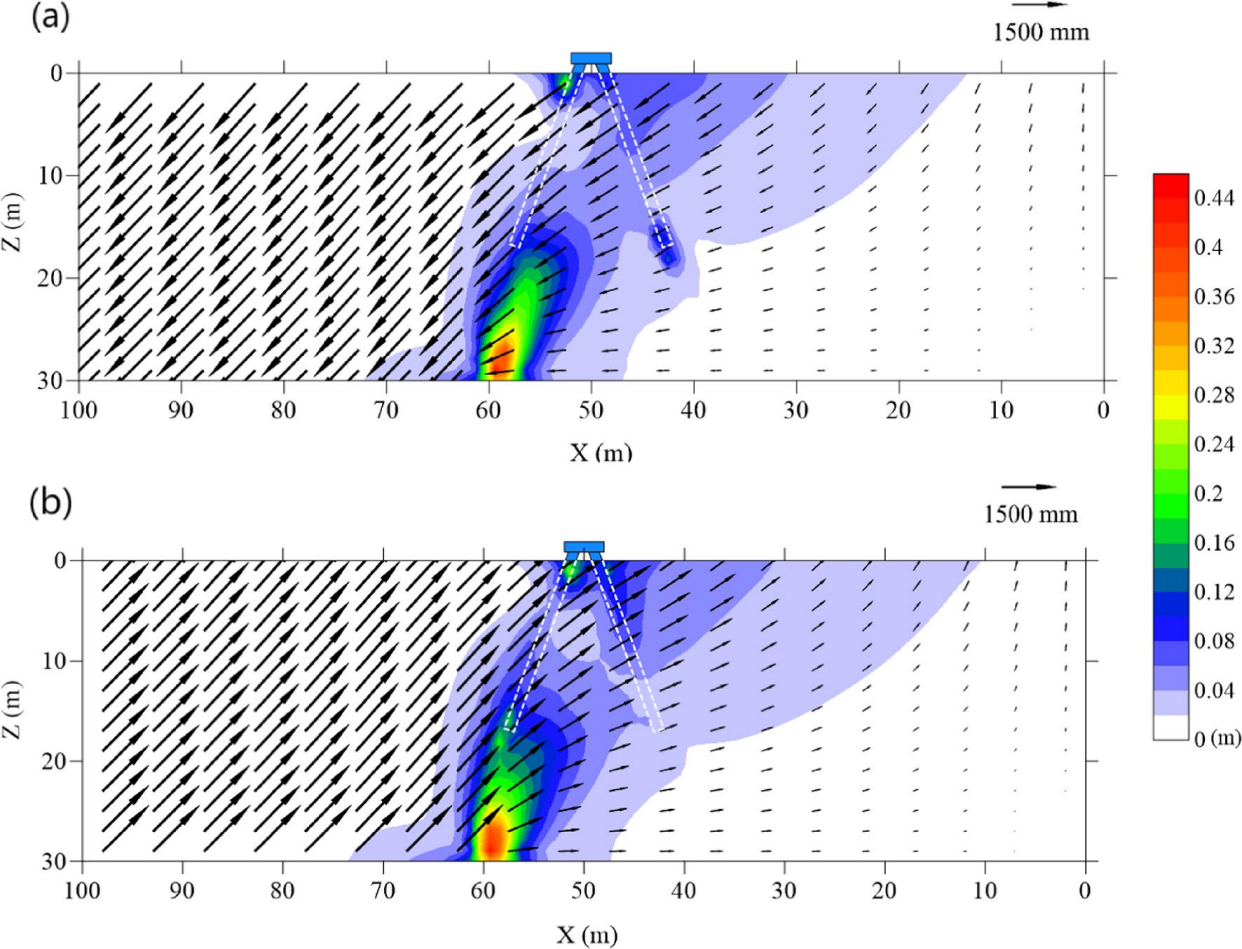
Soil displacement vectors and shear strain contours due to (**a**) Normal faulting (**b**) Reverse faulting.

## Interpretation of computed results

### Induced soil movement and fault propagation

Figure 5a and b illustrate the displacement and shear strain distribution in the soil under normal and reverse faulting scenarios, respectively. Significant shear strain is observed in the bottom soil layer, which corresponds to the starting point of the fault plane. This strain arises from the relative dislocation of the hanging wall and the footwall. The intensity of shear strain diminishes as the fault plane extends upward. Overall, the dislocation of the hanging wall generates shear strain distributed across a large region surrounding the vertical projection of the bedrock fault plane and the batter pile foundation. This highlights the considerable influence of soil shear forces on the behavior of the batter pile foundation. The differential settlement induced by faulting results in uneven ground movement, tilting the pile cap and redistributing loads within the pile group. In normal faulting, settlement near the hanging wall increases axial forces in pile P2, leading to greater penetration and pile deflection. For reverse faulting, the uplift caused by the hanging wall results in oblique forces that shift additional loads to pile P1, creating significant bending and deflection along its length. The redistribution of loads during these scenarios alters the shaft resistance of individual piles. For instance, normal faulting increases shaft friction near pile P2, while reverse faulting enhances load transfer to pile P1 due to oblique soil displacement. These changes in load transfer mechanisms, coupled with localized shear strain around the pile toes and along the pile bodies, affect the overall structural stability and performance of the foundation system.

There are obvious differences in the shear strain patterns induced by normal and reverse faulting. During normal faulting, the movement of the hanging wall causes the pile cap to tilt, increasing the axial force on pile P2. Due to insufficient end-bearing capacity pile P2 is forced deeper into the soil, generating significant shear strain around its toe. Conversely, during reverse faulting, shear strain is concentrated around the toe of pile P1. As the pile cap tilts further, more working load is transferred to pile P1. This, combined with the oblique and upward movement of the surrounding soil, increases the axial force on pile P1. The insufficient end-bearing capacity ultimately results in the failure of the soil structure around the toe of pile P1, causing the shear strain to propagate from the bottom soil layer to the toe of pile P1.

Regarding soil displacement, it is worth noting that the soil movement around pile P2is almost perpendicular to the pile body under both normal and reverse faulting conditions. In contrast, oblique soil displacement is observed along the body of pile P1. This displacement increases the shaft resistance of pile P1, which subsequently affects its axial force.

### Induced pile cap displacement and tilting

**Fig. 6:**
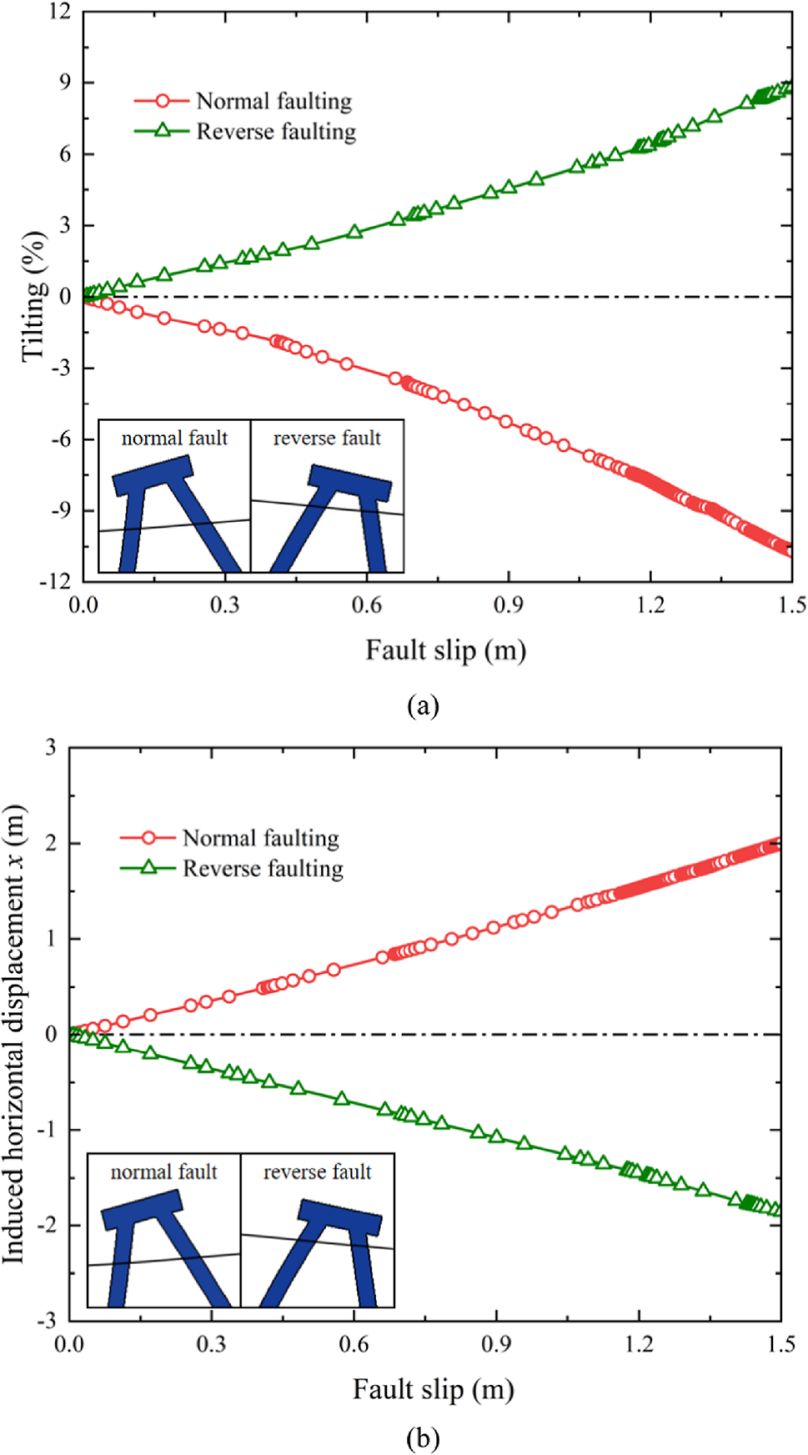
Induced pile cap movement due to normal and reverse faulting: (**a**) tilting (**b**) horizontal displacement.

reverse fault ruptures is illustrated in Fig. 6a. A positive tilt value indicates movement toward the direction of pile P1. As shown in the figure, the pile cap tilts in opposite directions during normal and reverse faulting due to the opposing directions of fault slip. For the same fault slip distance, the reverse fault lifts the pile foundation by 0.79 m, which is less than the 0.94 m settlement caused by the normal fault. Additionally, the tilting of the pile cap induced by the normal fault reaches 10.7%, which is more severe than the 8.8% tilt observed during reverse fault development. Furthermore, the rate of tilt increases progressively as the fault slip grows. This phenomenon is primarily attributed to the vertical working load applied to the pile cap. As the tilting develops, the distribution of the working load between pile P1 and pile P2becomes increasingly uneven. For instance, during normal faulting, the tilting of the pile cap causes a greater proportion of the load to shift toward pile P1, reducing the load borne by pile P2. This uneven load distribution results in differential settlement between pile P1 and pile P2, further amplifying the tilting effect.

The tilting behavior can be better understood in the context of displacement and shear strain contours discussed previously. The differential ground movement caused by normal faulting generates significant shear strain around the toe of pile P2, leading.

to its deeper penetration and contributing to the overall settlement of the pile group. Conversely, during reverse faulting, the upward movement of the hanging wall produces shear strain concentrated around the toe of pile P1, lifting the pile foundation and tilting the pile cap toward pile P2. These localized soil deformations directly influence the stability of the pile cap and exacerbate tilting as fault slip progresses.

Figure 6b presents the horizontal displacement of the pile cap during normal and reverse fault ruptures. A positive value indicates movement toward pile P1. As expected, the pile cap moves in opposite directions for normal and reverse faults, reflecting the directional nature of fault slip. The induced horizontal displacement increases linearly with the progression of fault slip, highlighting the close relationship between the movement of the hanging wall and the stability of the batter pile foundation. This linear trend also indicates that the stability of the pile foundation is heavily influenced by the magnitude of fault-induced horizontal soil displacements.

### Induced pile group deflection due to the development of fault ruptures

**Fig. 7:**
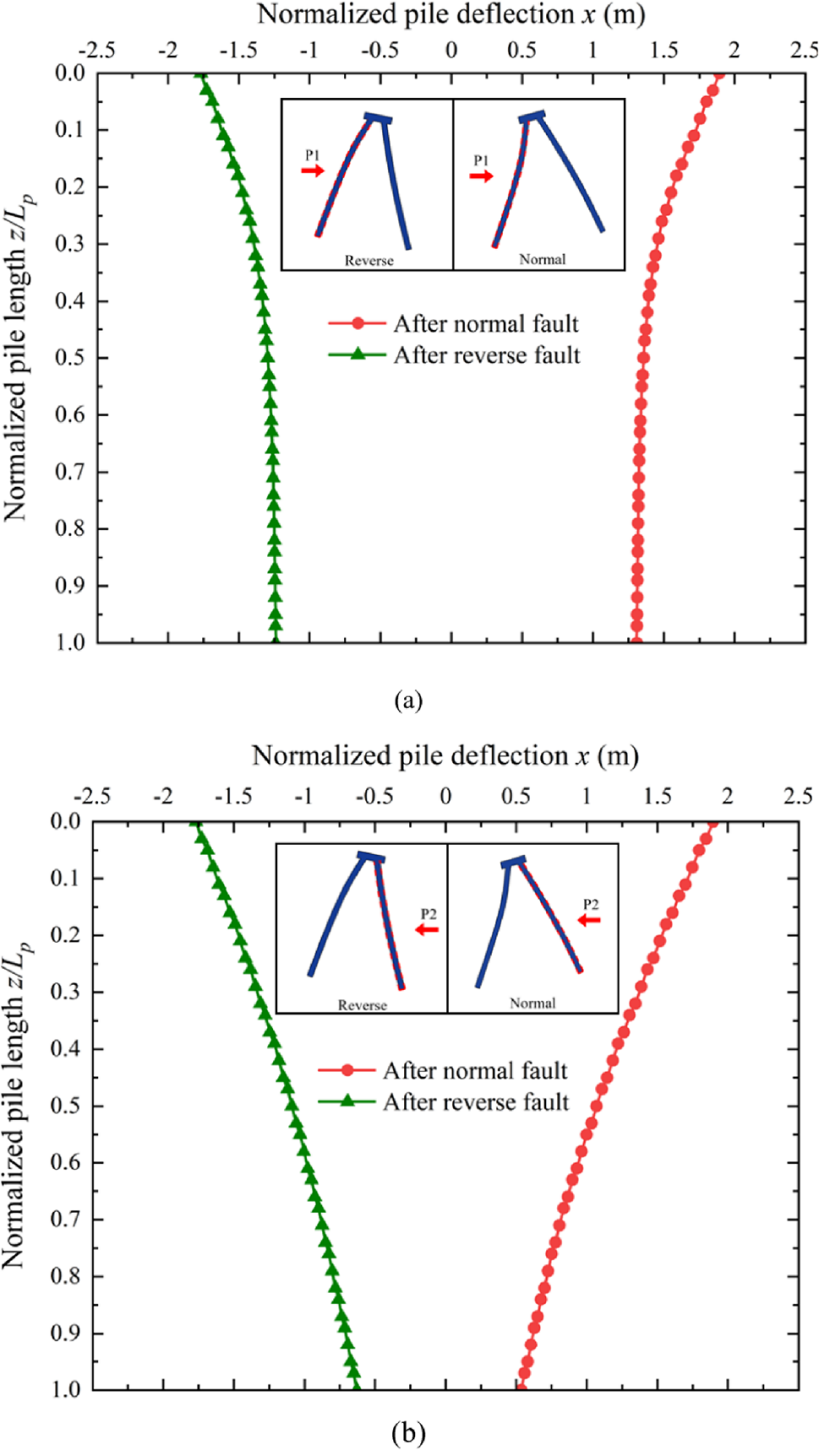
Normalized pile shaft deflection due to fault ruptures: (**a**) P1 (**b**) P2.

To better understand the deformation mechanism of the batter pile foundation under the development of normal and reverse fault ruptures, deflection profiles for pile P1 and pile P2 were derived from the numerical models, as shown in Fig. 7. The results indicate that the batter piles bend in opposite directions during normal and reverse faulting, with distinct deflection characteristics for pile P1 and pile P2 .

For pile P1, as illustrated in Fig. 7a, deflection is primarily concentrated in the upper region of the pile, near the ground surface. This localized bending changes the normal effective stress between the pile shaft and the surrounding soil in that region. The significant deflection observed in the upper portion suggests severe pile shaft damage, as substantiated by the stress and damage contours. This damage pattern is attributed to the oblique soil displacement along the body of pile P1, which maintains. shaft resistance in most of the pile but induces bending near the pile cap due to foundation tilting. Referring to Fig. 3, the tilting of the pile foundation causes a highlighted region of shear strain to appear near the ground surface around pile P1, marking a zone of concentrated deformation.

In contrast, the deflection profile for pile P2, as shown in Fig. 7b, reveals a more uniform bending along the entire length of the pile. The development of fault ruptures induces perpendicular soil displacement relative to pile P2, which bends the pile body evenly without creating significant localized concentrations of shear strain or internal forces. Consequently, damage to pile P2 is distributed evenly along the pile shaft, as opposed to the concentrated damage observed in pile P1.

The contrasting deflection patterns of pile P1 and pile P2 can be explained by the differing directions of surrounding soil movement. As observed in Fig. 5, the soil around pile P2 exhibits oblique displacement roughly aligned with the pile. This movement preserves the structural integrity of the soil near pile P1 but generates significant shear strain near the pile head due to the tilting of the pile foundation. This.

tilting leads to differential load distribution between pile P1 and pile P2, further increasing bending near the upper region of pile P1. Conversely, the soil around pile P2 moves perpendicular to the pile shaft, uniformly bending the pile without causing localized shear strain or damage.

This revealed that the deflection and damage profiles of pile P1 and pile P2 are directly influenced by the interaction between the foundation tilting, fault-induced soil displacement, and resulting shear strain. While pile P1 experiences concentrated bending near its upper region due to oblique soil movement and foundation tilting, pile P2 undergoes uniform deflection due to perpendicular soil displacement along its length.

### Damage condition of the batter pile foundation

The damage conditions and deformation mechanisms of the batter pile foundation under normal and reverse faulting are illustrated in Fig. 6. Both piles P1​ and P2 bent in the same direction during the fault rupture events, consistent with the tilting of the pile cap observed earlier. The batter pile foundations experienced significantly more tensile damage than compressive damage overall. At the junctions between the piles and the pile cap, severe damage occurred as anticipated^27,28^. However, critical tensile damage was evident, while no compression damage was observed at these junctions. For the pile shafts, tensile damage was predominantly distributed along the right-side surfaces of both batter piles, whereas compressive damage was localized to small regions on the left-side surfaces during normal fault rupture. Specifically, the tilting of the pile cap, as influenced by fault-induced displacement and shear strain, caused uneven loading on the piles, increasing tensile forces on one side while inducing limited compression on the opposite side.

Unexpectedly, during reverse fault rupture, no compressive damage was observed on pile P2, and the compressive damage on pile P1 was relatively slight. This difference in damage patterns can be attributed to the oblique upward movement of the hanging wall during reverse faulting, which redistributed forces and resulted in. lower compression on the pile shafts. These observations, combined with the damage conditions discussed above, suggest that the batter pile foundation is more vulnerable to failure due to the widespread tensile damage caused by both normal and reverse fault ruptures.

**Fig. 8:**
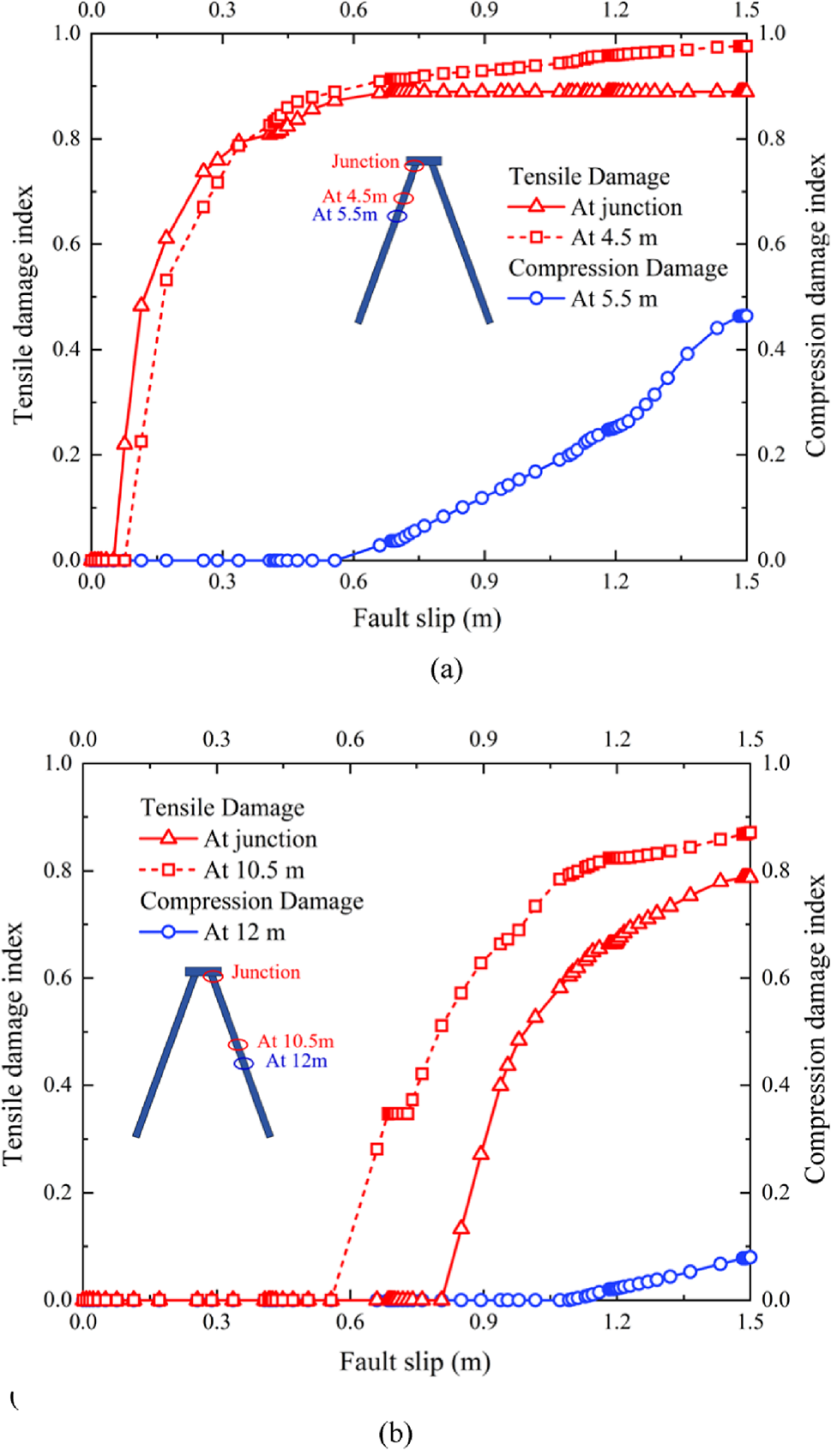
Progressive damage factors during occurrence of normal fault rupture in piles (**a**) P1 and (**b**) P2.

**Fig. 9:**
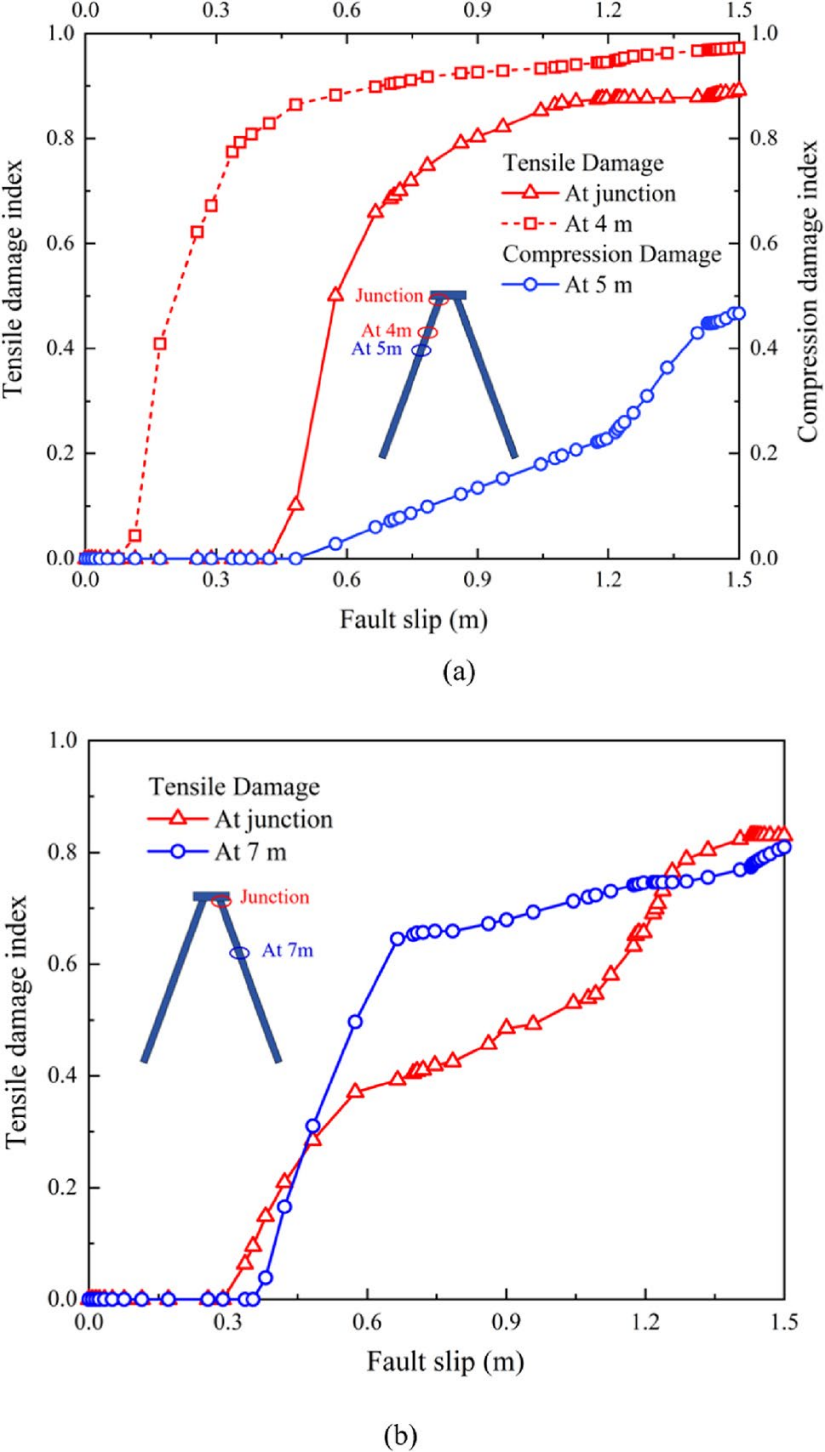
Progressive damage factors during occurrence of reverse fault rupture in piles (**a**) P1 and (**b**) P2.

The tilting of the pile cap, as shown in Figs. 6 and 7, is a critical factor influencing the damage distribution. The differential displacement and shear strain contours (Fig. 5) demonstrate the soil movement increases tensile forces on certain regions of the piles. For instance, the oblique displacement of soil near pile P1​ during normal faulting concentrates tensile forces on its right side, consistent with the observed damage. Meanwhile, the uniform soil movement around pile P2 induces a more distributed bending pattern, leading to tensile damage across its length but with minimal compressive damage. During reverse faulting, the upward movement of the soil reduces compression on both piles while still generating significant tensile stress, particularly at the junctions with the pile cap.

It is revealed the deformation and damage mechanisms of the batter pile foundation are strongly influenced by the interplay of fault-induced soil displacement, shear strain, and tilting of the pile cap. The widespread tensile damage, especially during normal faulting, highlights the vulnerability of the foundation to such seismic events, with the junctions and right-side pile surfaces being particularly critical regions.

### Normal effective stress along the pile induced by normal and reverse faults

The soil movement induced by fault ruptures significantly alters the normal effective stress distribution along the pile shaft, providing critical insights into soil-pile interaction mechanisms. The changes in normal effective stress along piles P1 and P2 after the development of normal and reverse fault ruptures are shown in Fig. 8a and b.

**Fig. 10:**
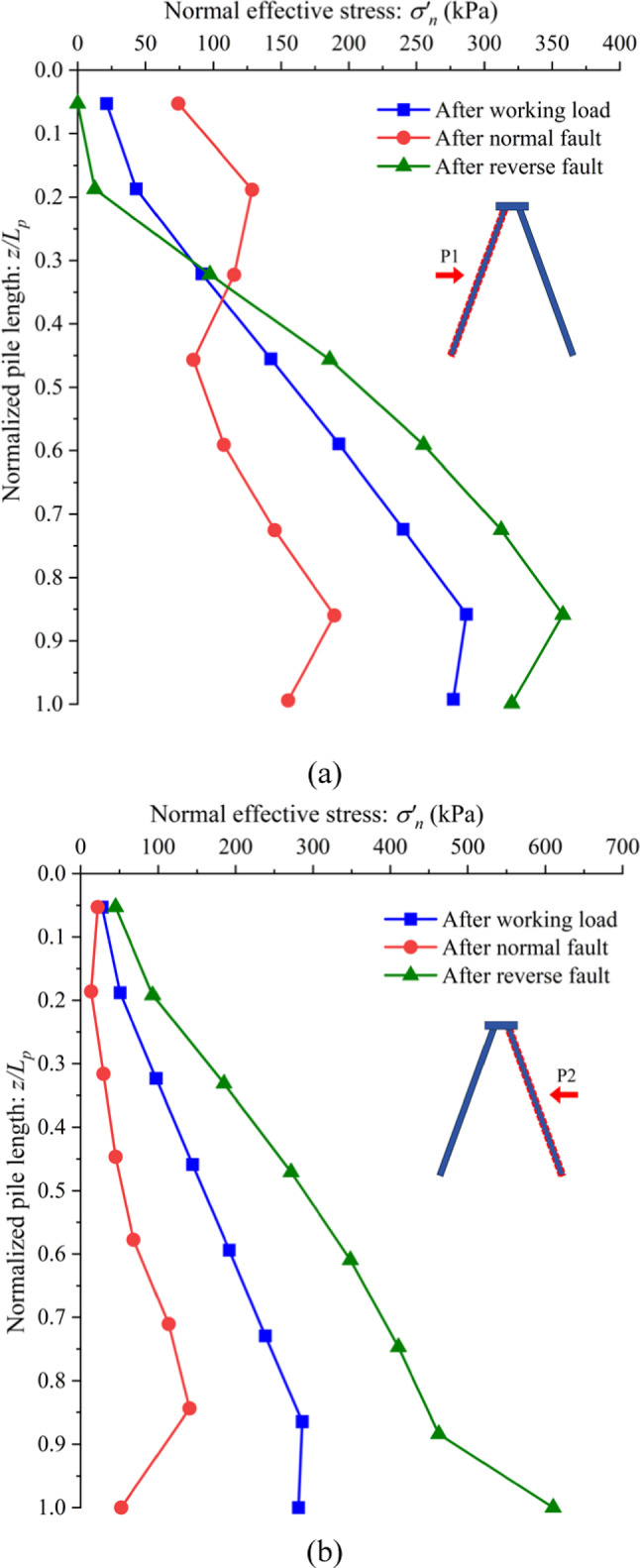
Change is normal effective stress distribution along the piles (**a**) P1 (**b**) P2 due to normal and reverse fault ruptures.

After the occurrence of the normal fault, the normal effective stress near the ground surface along pile P1 increases. This is because the deformation of pile P1 pushes the surrounding soil in the direction opposite to pile P2, causing resistance from the soil. This increased resistance near the upper shaft of pile P1 corresponds to the smaller bending moment observed in this region compared to the reverse fault scenario, as illustrated in Fig. 10a. Additionally, the tilting of the pile cap during normal faulting amplifies this stress redistribution, concentrating forces near the upper section of pile P1 where significant shear strain was observed in Fig. 5.

In the reverse fault scenario, the movement of the hanging wall enfolds the batter pile foundation, leading to an increase in normal effective stress along the lower shaft of pile P1 and across the entire shaft of pile P2. However, in the shallow region of pile P1, the soil movement aligns with the direction of the pile bending, resulting in a reduction of normal effective stress in this region. This reduction aligns with the observed contours of shear strain in Fig. 5, where localized deformation at the pile head is evident.

For pile P2, unlike pile P1, the deformation of the pile shaft is more uniform. Consequently, no significant changes in normal effective stress are observed near the upper region of pile P2. Instead, the normal effective stress varies uniformly along the length of pile P2 in response to soil movement induced by both normal and reverse faults, as shown in Fig. 10b. This uniform stress distribution is consistent with the overall bending mechanism of pile P2, where perpendicular soil displacement induces steady deformation along its entire length. The tilting of the pile cap, influenced by the differential displacement and shear strain patterns (Fig. 5), further accentuates these stress changes. In normal faulting, the tilting amplifies the load transfer toward P1, intensifying stress near its upper section, while pile P2 bore a more uniform load. During reverse faulting, the upward movement of the hanging wall redistributes forces across both piles, reducing localized stress concentrations but still increasing overall stress along the shafts.

### Axial force along the pile induced by normal and reverse faulting

**Fig. 11:**
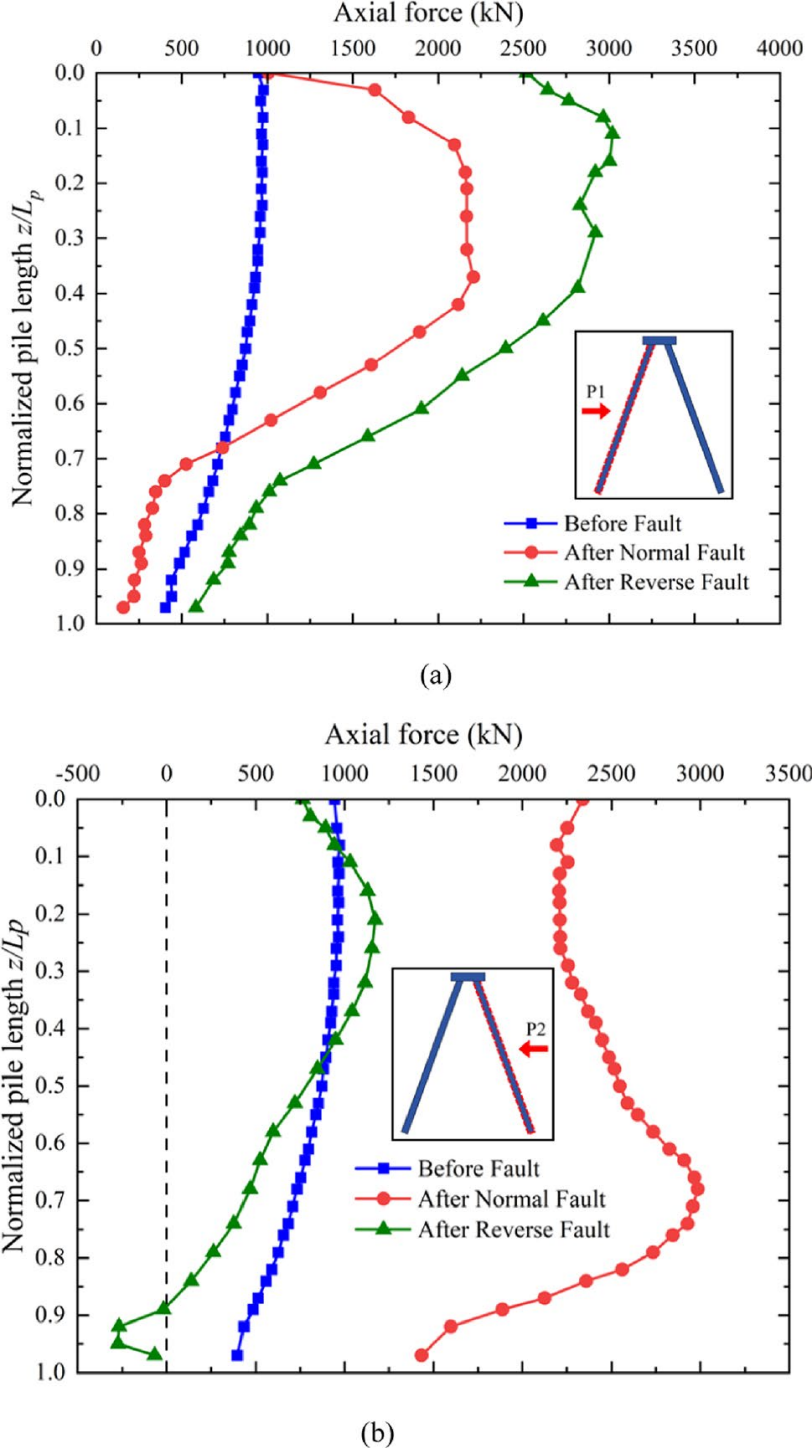
Changes in axial force distribution along the piles due to fault ruptures: (**a**) P1 (**b**) P2.

Figure 11a illustrates the development of axial force along pile P1 during normal and reverse faulting scenarios^32^. Under the applied working load, the axial force in pile P1 gradually decreases with depth**Error! Reference source not found.**. During the progression of normal faulting, pile P1 bends to the left, preventing the axial force from being fully transmitted to the pile tip. Instead, the force is partially redistributed to the surrounding soil, causing the axial force to first increase and then decrease along the pile depth. Overall, the axial force distributions along pile P1 during normal and reverse faulting are quite similar, except for the additional axial force at the pile tip caused by the upward movement of the surrounding soil during reverse faulting.

In contrast, the axial force distribution along pile P2 shows significant differences between normal and reverse faulting, as seen in Fig. 11b. During reverse faulting, there is no substantial increase in axial force along pile P2, unlike the pattern observed during normal faulting. This is because the uplifted pile P1 takes more of the working load from the pile cap, reducing the axial force transferred to pile P2. Peaks in axial force are observed at the bent regions of the pile shaft, similar to those seen in pile P1. Notably, normal faulting causes the most severely bent region to occur at deeper sections of the pile compared to reverse faulting, as shown in Fig. 11b. This is why the maximum axial force is observed at approximately 70% of the pile length, deeper than the bending regions associated with reverse faulting, which are located at around 20–30% of the pile length. Interestingly, the axial force at the toe of pile P2 becomes negative after deformation of the surrounding soil and structure. This indicates the development of a surface soil arching phenomenon around the pile toe^21^. This behavior highlights the complex interaction between pile deformation, soil movement, and load redistribution during fault rupture events.

### Bending moment along the pile induced by normal and reverse faulting

**Fig. 12:**
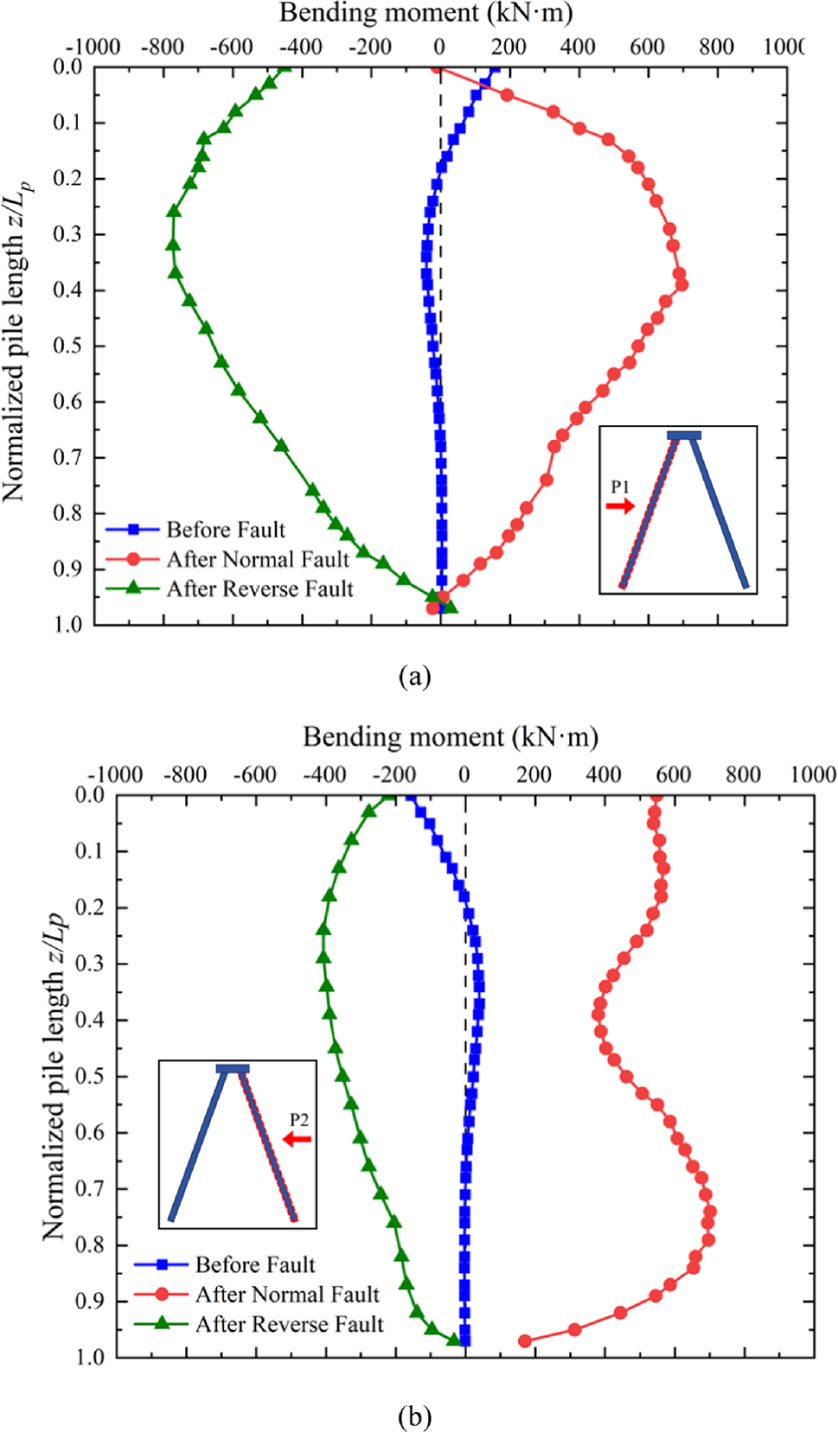
Induced bending moment distribution along the piles due to fault ruptures: (**a**) P1 (**b**) P2.

The bending moment distributions along the piles P1 and P2 are shown in Fig. 12, respectively. It can be observed that the maximum values of bending moment.

occurred in regions on the pile shafts. Under the applied working load, the maximum bending moment occurs at the junction between the pile and the cap. During the development of normal and reverse faulting, the bending moment along pile P1 increases and reaches its maximum at approximately 30% of the normalized pile length, as illustrated in Fig. 12a. Interestingly, the bending moment distribution pattern on P1 resembles that of batter pile groups subjected to lateral working loads^29^. Normal and reverse faults generate bending moments in opposite directions along the pile shafts, corresponding to the slipping directions of the hanging wall. For normal faulting, both piles bend to the left, while reverse faulting causes bending to the right. At the junctions between the piles and the pile cap, the bending moment can reverse direction due to soil movement, leading to severe tensile damage at these connections, as highlighted in Fig. 9.

### Shearing force along the pile induced by normal and reverse faulting

**Fig. 13:**
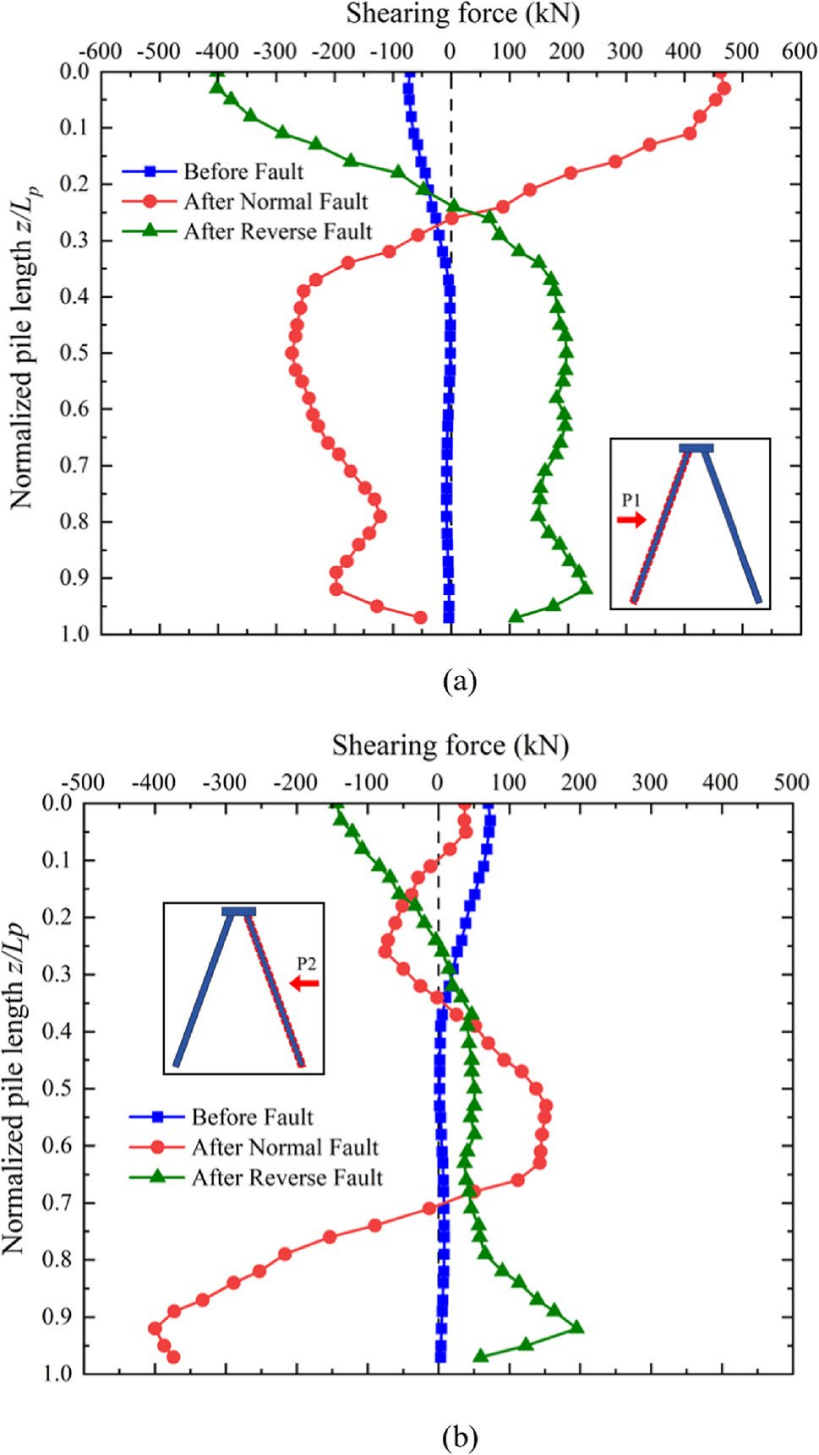
Induced shearing force distribution along the piles: (**a**) P1 (**b**) P2.

As previously discussed, significant deflection occurred in the upper region of the shaft of pile P1(refer to Fig. 7) after the development of normal and reverse faults. This deflection resulted in a large positive shear force in that area, transferred from the pile cap, as shown in Fig. 13a. The negative shear force observed was due to the constraint provided by the surrounding soil. Since the movement direction of the soil around pile P1 during both normal and reverse faulting was approximately along the pile shaft, the shear force induced by soil movement was minimal. Consequently, the shear force distribution along pile P1 was primarily governed by the bending deformation of the pile shaft, leading to symmetrical curves.

In contrast, the shear force distribution along pile P2 (Fig. 13b) was influenced by the direction of soil movement, which was nearly perpendicular to the pile shaft during fault propagation. In the case of reverse faulting, the soil moved in the same direction as the bending of pile P2, resulting in a shear force distribution similar to that of pile P1, but with reduced magnitude due to the constraint of the surrounding soil. However, during normal faulting, the soil movement opposed the bending direction of pile P2, causing a pronounced positive shear force between 50% and 70% of the pile length. This increase was induced by the perpendicular soil movement relative to the pile shaft. At the pile tip, the soil movement caused by normal faulting was minimal. The relative movement between the soil and the pile tip led to a negative shear force at the toe of the pile P2. This behavior highlights the varying contributions of soil movement and pile bending to the shear force distributions along the pile shafts.

## Practical implications and design recommendations for seismic regions

The findings of this study offer critical insights into the design and performance optimization of batter pile foundations under fault rupture scenarios. Specific engineering implications and actionable design recommendations are summarized as follows:

### Mitigation of tensile damage in batter piles

This study reveals that batter piles are particularly vulnerable to tensile damage resulting from differential loading and displacement induced by normal and reverse faulting. To address these vulnerabilities, several mitigation strategies are recommended. First, high-tensile-strength materials should be employed in the upper sections of pile shafts and at the junctions with the pile cap, as these areas are subjected to the highest tensile stresses during seismic events. Reinforcing these critical zones can significantly enhance the ability of the piles to resist tensile forces and improve overall structural integrity. Additionally, the design of pile-to-cap connections should be strengthened using ductile materials and energy-dissipating mechanisms. These enhancements will enable the connections to better accommodate the significant bending moments and shear forces observed during fault ruptures, thereby reducing the likelihood of damage and improving the resilience of the foundation system.

### Enhancing load distribution and reducing tilting

Fault-induced soil movements cause uneven load distribution among the piles, leading to cap tilting and structural instability. To address these issues, engineers can implement specific design strategies to enhance foundation performance. One effective approach is to modify the spatial arrangement of batter piles to optimize load sharing and minimize differential settlement, thereby improving the overall stability of the pile group. Additionally, increasing the rigidity of the pile cap can significantly reduce tilting by redistributing loads more evenly across the pile group. These measures collectively help mitigate the adverse effects of fault-induced movements, ensuring a more stable and resilient foundation system.

### Design adaptations for specific faulting scenarios

Normal faulting often leads to deeper penetration and localized damage in certain piles, posing significant stability challenges. To address this, implementing larger end-bearing pile tips can help distribute loads more effectively and reduce the risk of failure. Additionally, soil-improvement techniques near critical pile toes can further enhance stability by strengthening the surrounding soil and mitigating excessive deformation. In contrast, reverse faulting introduces uplift forces and oblique movements that can compromise the structural integrity of pile foundations. To counteract these effects, pile shafts can be pre-tensioned or equipped with anchor systems designed to resist upward displacements, ensuring greater resilience against.

### Recommendations for monitoring and maintenance

Regular inspection and monitoring of pile foundations in seismic regions are essential. The use of embedded sensors to track stress distribution and displacement can enable early detection of structural vulnerabilities, allowing for timely interventions.

## Conclusion

Based on the computed results, the following conclusions can be drawn:

1. Normal faulting caused greater pile cap displacement and tilting compared to reverse faulting. For the same fault slip distance, normal faulting resulted in a 10.7% tilt and 0.94 m of settlement, while reverse faulting caused an 8.8% tilt and 0.79 m of uplift. To mitigate such effects, pile cap designs should incorporate features to resist higher tilting forces, such as reinforced connections or distributed support mechanisms.
2. Tensile damage occurred earlier and was more severe than compressive damage under both reverse and normal fault ruptures. Reinforcing pile shafts and junctions with stronger steel bars or advanced composite materials would effectively enhance the structural resilience of batter pile foundations, especially against tensile stresses.
3. The most severely damaged areas were not limited to the joints between the piles and the pile cap but extended along the pile shafts. However, damage near the pile tips was relatively minor. This highlights the need for future research to investigate stress distribution and damage propagation along pile shafts to develop enhanced reinforcement strategies for these vulnerable regions.
4. All battered piles in the group bent in the direction of the hanging wall slip due to soil movement. Consequently, tensile damage occurred on one side of the pile bodies, while compression damage was limited to the opposite side. Consider asymmetrical reinforcement or protective measures to address the directional nature of fault-induced damage.
5. Pile P1 experienced significantly severe damage, with concentrated deflection in its upper shaft, a critical area for structural integrity. In contrast, Pile P2 exhibited more uniform deflection due to variations in soil movement around the piles. Reinforcement schemes should be tailored to the specific deflection profiles of individual piles, with priority given to the upper-shaft regions of piles like P1.

## Data Availability

“All data generated or analysed during this study are included in this published article.”
